# PPAR Agonists and Metabolic Syndrome: An Established Role?

**DOI:** 10.3390/ijms19041197

**Published:** 2018-04-14

**Authors:** Margherita Botta, Matteo Audano, Amirhossein Sahebkar, Cesare R. Sirtori, Nico Mitro, Massimiliano Ruscica

**Affiliations:** 1 Dipartimento di Scienze Farmacologiche e Biomolecolari, Università degli Studi di Milano, 20133 Milan, Italy; margherita.botta@unimi.it (M.B.); matteo.audano@unimi.it (M.A.); massimiliano.ruscica@unimi.it (M.R.); 2 Biotechnology Research Center, Pharmaceutical Technology Institute, Mashhad University of Medical Sciences, Mashhad 9177948564, Iran; amir_saheb2000@yahoo.com; 3 Neurogenic Inflammation Research Center, Mashhad University of Medical Sciences, Mashhad 9177948564, Iran; 4 School of Pharmacy, Mashhad University of Medical Sciences, Mashhad 9177948564, Iran; 5 Centro Dislipidemie, Azienda Socio Sanitaria Territoriale Grande Ospedale Metropolitano Niguarda, 20162 Milan, Italy; cesare.sirtori@icloud.com

**Keywords:** metabolic syndrome, PPARs, pemafibrate, elafibrinor

## Abstract

Therapeutic approaches to metabolic syndrome (MetS) are numerous and may target lipoproteins, blood pressure or anthropometric indices. Peroxisome proliferator-activated receptors (PPARs) are involved in the metabolic regulation of lipid and lipoprotein levels, i.e., triglycerides (TGs), blood glucose, and abdominal adiposity. PPARs may be classified into the α, β/δ and γ subtypes. The PPAR-α agonists, mainly fibrates (including newer molecules such as pemafibrate) and omega-3 fatty acids, are powerful TG-lowering agents. They mainly affect TG catabolism and, particularly with fibrates, raise the levels of high-density lipoprotein cholesterol (HDL-C). PPAR-γ agonists, mainly glitazones, show a smaller activity on TGs but are powerful glucose-lowering agents. Newer PPAR-α/δ agonists, e.g., elafibranor, have been designed to achieve single drugs with TG-lowering and HDL-C-raising effects, in addition to the insulin-sensitizing and antihyperglycemic effects of glitazones. They also hold promise for the treatment of non-alcoholic fatty liver disease (NAFLD) which is closely associated with the MetS. The PPAR system thus offers an important hope in the management of atherogenic dyslipidemias, although concerns regarding potential adverse events such as the rise of plasma creatinine, gallstone formation, drug–drug interactions (i.e., gemfibrozil) and myopathy should also be acknowledged.

## 1. Introduction

The incidence of metabolic syndrome (MetS), representing a global public health issue, has been estimated to vary from 20 to 27% in developing countries [1,2] to 35% in the USA [3]. MetS is a cluster of cardiometabolic risk factors, from high triglycerides (TGs), to elevated waist circumference (WC), high blood pressure (BP) and insulin resistance [4].

Following the first definition of MetS by the World Health Organization (WHO), several expert panels attempted to introduce stricter diagnostic criteria. In 2001, the National Cholesterol Education Program (NCEP) Adult Treatment Panel III (ATP III) [5] recognized that the clustering of the metabolic risk factors included in the syndrome were indeed cardiovascular (CV) risk factors. In 2003, the American Association of Clinical Endocrinologists (AACE) modified the ATP III criteria highlighting the central role of insulin resistance in the pathogenesis of the syndrome [6]. In 2005, the International Diabetes Federation (IDF) issued a consensus document aimed at introducing a clinically useful definition of MetS in order to identify individuals at high risk of CV disease (CVD) and type 2 diabetes mellitus (T2D) on a worldwide basis [7]. In the same year, the American Heart Association (AHA)/National Heart, Lung and Blood Institute (NHLBI) suggested more specific criteria for the diagnosis of MetS [7]. Finally, a joint statement of IDF, NHLBI, AHA, World Heart Federation and International Association for the Study of Obesity, best known as the “Harmonization definition”, has been introduced and now represents the most commonly recognized criterion for the clinical diagnosis of MetS [4] (Table 1).

Carriers of MetS are at higher risk of developing atherosclerotic CVD, a condition worsened by the so called “atherogenic dyslipidemia”. This mixed dyslipidemia has emerged as the most clinically relevant “competitor” of elevated low-density lipoprotein cholesterol (LDL-C) among lipid risk factors. It is characterized by hypertriglyceridemia, low high-density lipoprotein (HDL)-cholesterol levels, and the prevalence of small, dense low-density lipoprotein (LDL) particles as well as an accumulation of cholesterol-rich remnant particles [9].

In this metabolic derangement, peroxisome proliferator-activated receptors (PPARs), i.e., nuclear receptors involved in the regulation of metabolic homeostasis, represent a valuable therapeutic target. PPAR activators have provided significant benefit in patients with primary hypertriglyceridemia (i.e., fibrates and omega-3 fatty acids, both PPAR-α agonists), as well as in cases of mixed hyperlipidemias with raised TGs and low HDL-C; conversely, PPAR-γ activators have become choice drugs in T2D.

PPAR agonists are generally recognized as effective pharmacological tools for the management of MetS [10,11]. A growing interest in PPAR activators has been acknowledged in recent years as they have been used in the more and more frequent occurrence of non-alcoholic fatty liver disease [12], the hepatic manifestation of MetS. However, post-marketing adverse effects should be recalled [13], i.e., weight gain, fluid retention, congestive heart failure, liver and gallbladder disease, renal effects, bone fractures, myopathy and rhabdomyolysis; these two last particularly with fibrates with an added risk when co-administered with statins [14,15].

Hence, the present review was aimed at discussing available evidence on new PPAR agonists in the clinical setting as well as at describing molecular mechanisms underlying the effects of these drugs. This review also attempts to reduce the current disagreement on the interpretation of outcomes of clinical trials with fibrates. To this end, we have revised and updated the available English-language studies relevant to the key clinical questions, published up to April 2018.

## 2. Peroxisome Proliferator-Activated Receptor: Key Players in Energy Homeostasis

PPARs are a subfamily of three ligand-inducible transcription factors, belonging to the superfamily of nuclear hormone receptors. In mammals, three different isoforms of PPARs have been described so far: PPAR-α, PPAR-β/δ and PPAR-γ. PPARs belong to the nuclear hormone receptor superfamily and, by binding to PPAR-responsive regulatory elements (PPRE), heterodimerise with the retinoid X receptor (RXR) and control a group of genes involved in adipogenesis, lipid metabolism, inflammation and maintenance of metabolic homeostasis [16,17,18,19,20]. PPAR-α is the first identified member and is mainly expressed in energy-demanding tissues that show high rates of β-oxidation (i.e., liver, kidney, heart and muscle). On the other hand, PPAR-β/δ is ubiquitously expressed in humans, whereas in mice it is expressed to a higher extent in the gastrointestinal duct, specifically stomach, large and small intestine. PPAR-γ is expressed at high levels in the adipose tissue.

PPARs are activated by fatty acids and eicosanoids [21], as well as by small molecules, such as fibrates for PPAR-α, GW501516, GW0742, bezafibrate and Telmisartan for PPAR-β/δ, and glitazones for PPAR-γ. PPAR-α mediates the hypolipidemic function of fibrates in the treatment of hypertriglyceridemia and hypoalphalipoproteinemia [22], being the main regulator of intra- and extracellular lipid metabolism. Indeed, fibrates downregulate hepatic apolipoprotein C-III (ApoCIII) and stimulate lipoprotein lipase gene expression, thus being key players in TG metabolism [23].

**Figure 1:**
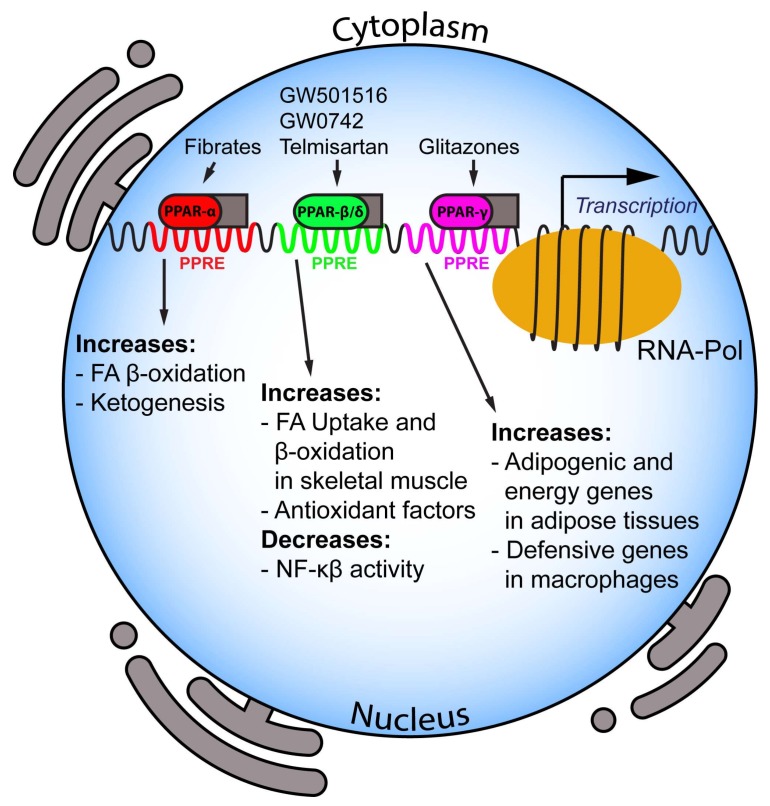
Major roles of different peroxisome proliferator-activated receptors (PPARs) isotypes. PPARs are a class of nuclear transcription factors that heterodimerize with retinoid X receptor (RXR, gray boxes) upon physiological (i.e., fatty acids) and synthetic activation (i.e., fibrates, glitazones etc.) to regulate the specific indicated pathways. FA, Fatty Acids; NFκB, Nuclear Factor-κB.

Moreover, PPAR-α activation raises plasma HDL-C via induction of hepatic apolipoprotein A-I and apolipoprotein A-II expression in humans. On the other hand, glitazones exert hypotriglyceridemic activity by activating PPAR-γ, in turn inducing lipoprotein lipase expression in adipose tissue [23]. Finally, PPARs exert their function on intracellular lipid metabolism by regulating key proteins involved in the conversion of fatty acids to acyl-CoA esters, fatty acid import into mitochondria and peroxisomal and mitochondrial fatty acid oxidation [24,25]. Major roles and functions of the PPAR isotypes are depicted in Figure 1.

### 2.1. PPAR-α

PPAR-α (also called NR1C1) activation occurs mainly under energy deprivation. This leads to the upregulation of intracellular energy metabolism, ultimately inducing ATP production from oxidative phosphorylation. PPAR-α mRNA is upregulated in mouse liver during fasting, whereas PPAR-α knock-out (KO) fasted mice display significant hypoglycemia, hypoketonemia, hypothermia, and increased plasma free fatty acids, thus suggesting an inhibition of fatty acid uptake and oxidation [26]. PPAR-α-mediated fatty acid catabolism is crucial for the synthesis of several metabolites to be used as energy sources by other tissues such as ketone bodies in the brain [27]. Classical genes regulated by this nuclear receptor are the β-oxidative enzymes, e.g., carnitine palmitoyltransferase 1A and 2 (*CPT1A* and *2*), acyl-CoA dehydrogenase very long chain (*ACADVL*), hydroxyacyl-CoA dehydrogenase trifunctional multienzyme complex subunit-α (*HADHA*); similarly, important ketogenic genes like 3-hydroxy-3-methylglutaryl-CoA synthase 2 (*HMGCS2*), 3-hydroxymethyl-3-methylglutaryl-CoA lyase (*HMGCL*) and acetyl-CoA acetyltransferase 1 (*ACAT1*) are stimulated by PPAR-α. This last seems to control liver glucose metabolism as well. Administration of fenofibrate to mice decreases expression levels of glucokinase and flux through this enzyme, suggesting a lower liver glucose uptake upon PPAR-α activation [28]. In another study, PPAR-α was found to induce pyruvate dehydrogenase kinase 4 (*PDK4*) expression, suppressing pyruvate transition to acetyl-CoA. Conversely, PPAR-α KO pups displayed a primary defect in gluconeogenesis, specifically from glycerol, leading to significant hypoglycemia [29].

PPAR-α induction also exerts an anti-inflammatory activity in mouse models, even though contrasting data are reported. The first evidence of PPAR-α involvement in the regulation of inflammation was provided by the group of Wahli more than 20 years ago [30]. The authors demonstrated that leukotriene B4 acts as a ligand for PPAR-α transcription, and the inflammatory response is prolonged in PPAR-α KO mice [31]. More recent studies have confirmed this association, showing that PPAR-α activation in mouse liver downregulates the CCAAT/enhancer binding protein β (C/EBPβ) as well as alpha (C/EBPα) and nuclear factor-κB (NFκB) protein expression, leading to lower levels of C-reactive protein, interleukin-6 and prostaglandins [32]. Conversely, dietary treatment with PPAR-α agonists increased lipopolysaccharide-induced plasma tumor necrosis factor α (TNF-α) levels that is instead reduced in PPAR-α-deficient mice, highlighting a possible role for PPAR-α also as a pro-inflammatory factor [33].

### 2.2. PPAR-β/δ

PPAR-β/δ (also called NR1C2) is the least well characterized isotype among PPARs. Nevertheless, it plays an important role in the metabolic adaptation of numerous tissues to environmental stimuli [34]. Physiologically, this isotype is activated by long-chain fatty acids, saturated and unsaturated, and by prostacyclin [35,36]. Notably, specific PPAR-β/δ activation leads to increased levels of fatty acid β-oxidation [37]. Moreover, PPAR-β/δ expression is upregulated specifically in skeletal muscle during fasting. These data were confirmed in PPAR-β/δ agonist treated L6 rat myocytes, showing increased fatty acid uptake and β-oxidation compared to controls [37].

Further studies suggest that PPAR-β/δ and physical exercise are tightly related; indeed, endurance training (6 weeks) boosted an up to 2.6-fold PPAR-β/δ protein expression in the tibialis anterior muscle [38]. The indispensable role of PPAR-β/δ in cell energy metabolism has been also shown in rat breast adenocarcinoma cells [39]. Specifically, the authors demonstrated that high PPAR-β/δ protein was associated with increased cancer cell growth in vitro and in vivo, suggesting that PPAR-β/δ favors breast cancer cell survival by regulating specific metabolic pathways.

PPAR-β/δ ligands have also been proposed as potential anti-inflammatory drugs [40,41]. Pharmacological activation of PPAR-β/δ in endothelial cells is associated with a potent anti-inflammatory effect, possibly by involving antioxidative genes and release of nuclear corepressors [42]. Moreover, investigation of PPAR-β/δ role in the modulation of NF-κB-driven inflammatory response confirmed the anti-inflammatory activity of this isotype [43]. The highly selective PPAR-β/δ agonist GW0742 in rats effectively antagonized lethality consequent to cecal ligation and puncture: drug treated animals had reduced release of pro-inflammatory cytokines and neutrophil infiltration in lung, liver, and cecum.

### 2.3. PPAR-γ

PPAR-γ (also called NR1C3) is mainly expressed in adipocytes (brown and white) and plays a major role in cell differentiation and energy metabolism [44,45]. Upregulation of PPAR-γ activity in vivo leads to bone loss and higher bone marrow adiposity, whereas downregulation leads to elevated bone mass [46]. The PPAR-γ agonists thiazolidinediones (TZDs) have a hypoglycemic action in ob/ob mice and improve insulin action in several models of obesity and diabetes [47,48]. A high correlation between the hypoglycemic activity of TZDs and their affinity for PPAR-γ has been repeatedly shown. While PPAR-γ-KO animals show embryonic lethality dying at 10.5–11.5 days postcoitum due to placental dysfunction, PPAR-γ heterozygote KO are characterized by higher insulin sensitivity and resistance to high-fat diet-induced insulin resistance [49,50,51].

PPAR-γ Pro12Ala partial loss-of-function mutation in humans leads to decreased body mass index, higher insulin sensitivity and protection from T2D [52]. The most accepted hypothesis is that PPAR-γ facilitates energy storage after high-fat diet challenge, partly by suppressing leptin expression in adipocytes [49]. In addition, PPAR-γ directly binds the promoter of almost all adipogenic genes, such as factors involved in glucose and fatty acid metabolism, indicating that this isotype is fundamental for the activation of metabolic programs during adipogenesis [53]. While PPAR-γ is not needed for macrophage differentiation, it is fundamental for a proper anti-inflammatory activity in adipose tissue macrophages [54]. This specific feature of PPAR-γ is due to cell type specific DNA binding, as shown in macrophages, where PPAR-γ cooperates mainly with the spleen focus forming virus (SFFV) proviral integration into the oncogene transcription factor [55]. On the other hand, it is well established that CCAAT/enhancer binding protein (C/EBP) α and β transcription factors are major partners of PPAR-γ during adipocyte differentiation [53].

Comparative analysis of PPAR-γ binding patterns in adipocytes and macrophages indicates that it binds to immune defense genes in macrophages, whereas the only cluster of genes shared with adipocytes is the one encoding metabolic factors [55]. Notably, myeloid lineage-specific PPAR-γ KO mice display lower alternative macrophage activation and a strong metabolic phenotype, characterized by diet-induced obesity, insulin resistance, and glucose intolerance [55]. In addition, PPAR-γ activation decreases T-lymphocyte-dependent inflammation of adipose tissue and development of insulin resistance in diet-induced obese mice [56]. Recently, it has been demonstrated that the PPAR-γ agonist pioglitazone may prevent or delay aortic aneurysm progression in patients [57]. Treated patients show decreased macrophage infiltration into the retroperitoneal periaortic fat, as well as tumor necrosis factor α (*TNFα*) and matrix metallopeptidase 9 (*MMP9*) gene expression. On the other hand, treatment increased adiponectin expressions in both tissues compared to controls. Neurons are protected by pioglitazone treatment [58]; indeed, neuron and axon susceptibility to both nitric-oxide donor-induced and microglia-derived nitric oxide-induced toxicity are reduced, whereas catalase expression is raised [58].

## 3. PPAR-α: Fibrates and Omega-3 Fatty Acids in the Metabolic Syndrome

The characteristic features of MetS, i.e., increased triglyceridemia, abdominal obesity, reduced HDL-C levels and increased glycemia, in addition to raised blood pressure, clearly indicate that PPAR-α agonists have an ideal profile to control most of these features [59].

### 3.1. Fibrates

The role of fibrates in the clinical management of disorders characterized by elevated TGs is now well established [60]. Fibrates, activators of the PPAR system, mainly PPAR-α, have shown significant benefit in clinical trials of CV prevention, i.e., reducing the occurrence of nonfatal myocardial infarction, particularly when restricting evaluation to patients with concomitant TG elevation and HDL-C reduction [61].

Clinical findings, at times disappointing (FIELD—Fenofibrate Intervention and Event Lowering in Diabetes—study) [62], have been hampered by inappropriate patient selection and, possibly, by the use of non-optimal drug formulations [63,64]. On the other hand, long-term re-evaluation of most recent studies, particularly the ACCORD—Action to Control Cardiovascular Risk in Diabetes—trial with fenofibrate, has clearly indicated that the agent has a heterogeneous response, but may have a valid indication in reducing CVD in appropriately selected patients, i.e., those with diabetes, hypertriglyceridemia and low HDL-C [65]. In these subjects, a definite benefit on the CV outcomes may be predicted.

When projected to the growing field of lipid-lowering treatments in clinical practice, these observations have fostered the development of newer pharmacological approaches, partially based on the PPAR target, but providing additional or alternative mechanisms which may lead to lipoprotein changes and altered glycemic parameters, not found with the established agents. Hence, this pharmacological approach may be of potential value in the clinical approach to the more and more frequent occurrence of MetS [66].

A recent meta-analysis from 22 RCTs involving a total of 11,402 subjects showed that fibrate administration decreased fasting plasma glucose (−5 mg/dL), insulin levels (−0.56 µIU/mL), and insulin resistance (HOMA-IR: −1.09), but not HbA1C. This latter evidence may be due to the short-term duration—less than three months—of the included studies. Moreover, the reduction in glucose, although statistically significant, was small and, thus, not clinically relevant [67]. These findings have been well characterized from studies with fenofibrate and bezafibrate. This latter, differently from the most selective PPAR-α modulator fenofibrate, acts as a pan PPAR activator for all three PPAR isoforms (α, γ and δ) [68]. Bezafibrate, similar to the selective PPAR-γ agonists, i.e., thiazolidinedione (TZDs) that are specifically used in T2D treatment, may exert a glucose-lowering activity but apparently without causing water retention, weight gain and peripheral edema that are potential side effects of glitazones [69,70]. Bezafibrate, compared with other fibrates, reduces the incidence of T2D, with a better reduction of blood glucose, HbA1C and insulin resistance [71].

#### Fibrates: Evidence from the Most Recent Clinical Trials

Pemafibrate (formerly known as K-877) is one of the newest members of the selective PPAR-α modulators, being >2000-fold more selective for PPAR-α vs. either PPAR-γ or -δ (delta) [72]. Pemafibrate has been recently approved in Japan for the treatment of hyperlipidemias, with a recommended dosage of 0.1 mg bid with the possibility of reaching a maximum of 0.2 mg bid [73].

The long-term efficacy of pemafibrate has been recently reported in a phase 3 multicenter, placebo-controlled, randomized, double-blind, parallel-group study (JapicCTI-142412) on T2D patients (HbA1c ≥ 6.2%) with fasting TGs ≥ 150 mg/dL, not on statins. The primary endpoint was the percentage change in fasting TG levels from baseline and secondary endpoints were changes in fasting and postprandial lipoproteins and glycemic parameters. Among the 167 eligible participants (mean age 60.5 years), 54 and 55 were randomized to pemafibrate 0.1 or 0.2 mg bid, respectively, and 57 to a placebo. Twenty-four weeks of treatment led to a significant 45% decrement of TGs regardless of dose; fasting TGs ≤ 150 mg/dL were achieved by 81.5% and 70.9% of patients on 0.2 and 0.4 mg/day, respectively; statistically significant when compared to the placebo group. In addition, non-HDL-C, cholesterol remnants, ApoB100, ApoB48, and ApoCIII levels were reduced with a concomitant rise of HDL-C and ApoA-I (Table 2) [74]. The reduction of ApoCIII levels confirmed the general activity of PPAR activators on this variable, in line with what reported with statin treatments [75].

Notably, pemafibrate led to a more anti-atherogenic profile, i.e., higher levels of medium, small and very small HDL particles vs very large and large particles at baseline. Although no changes were seen in LDL-C levels, a significant increment of large LDL and a reduction of small and very small particles were found in the pemafibrate arms. Modest changes were seen in glycemic parameters: only the 0.2 mg dose significantly reduced the HOMA-insulin resistance score with no significant changes in fasting glucose, insulin, glycated albumin and HbA1c. Both pemafibrate doses significantly raised circulating levels of FGF-21 [74]. All groups displayed comparable rates of adverse events and drug reactions, i.e., serum creatinine and liver enzyme increases.

The efficacy of pemafibrate over that of fenofibrate was reported in a 24-week, randomized, double blind, active-controlled, phase 3 trial. Patients with fasting TG ≥ 150 mg/dL as well as HDL-C ≤50 mg/dL for men and ≤55 mg/dL for women were randomly assigned to pemafibrate 0.1 (*n* = 73) or 0.2 (*n* = 74) mg bid or to fenofibrate 106.6 mg qd (*n* = 76). The primary efficacy analysis, i.e., percent change in fasting TG from baseline, demonstrated that pemafibrate treatments reduced TGs around −46% vs. −39.7% for fenofibrate. These findings can be translated into a further −6.5% and −6.2% difference in TG reduction for patients on 0.2 and 0.4 mg/day pemafibrate vs. fenofibrate. TC, non-HDL-C, ApoB, and VLDL-C were significantly decreased, and HDL-C, ApoAI, and ApoAII increased by both agents with no significant differences among treatment groups. Conversely, FGF-21 levels were raised to a greater extent in the 0.4 mg/day pemafibrate group vs. fenofibrate [76] (Table 2). Adverse drug reactions, such as rises in liver enzymes and serum creatinine, were observed in the fenofibrate group, not in the pemafibrate groups.

The non-inferiority of pemafibrate over fenofibrate was confirmed in a 12-week phase 3 trial enrolling 489 patients with TG ≥ 200 mg/dL and HDL-C ≤ 50 mg/dL. The TG lowering effects of pemafibrate were dose dependent −46.3% (0.1 mg/day), −46.7% (0.2 mg/day) and −51.8% (0.4 mg/day) and non-inferior to those of fenofibrate, −38.3% (100 mg/day) and −51.5% (200 mg/day) (Table 2). Adverse events were less frequent than with fenofibrate 200 mg/day [77].

The long-term efficacy of pemafibrate to treat residual hypertriglyceridaemia during statin treatment has been recently evaluated in two randomized, double-blind, placebo-controlled phase 2 trials. The primary endpoint was the percentage changes in fasting TGs from baseline [78]. The first trial study enrolled 188 patients with residual dyslipidemia (fasting TGs from 347 to 382 mg/dL) on a pitavastatin background with LDL-C in the range of 116–125 mg/dL. The 12-week pemafibrate administration (0.1, 0.2 or 0.4 mg/day) significantly reduced TG levels by −46.1%, −53.4% and −52%, respectively. Conversely, no TG reduction was observed in the pitavastatin monotherapy group. Combination therapy led to a significant rise of HDL-C (range: +12.7–19.7%), ApoAI (range: +1.5–6.6%) and ApoAII (range: +18.5–27.6%) and a reduction of non-HDL-C (range: −10.7–13.1%) and ApoB (range: −7.9–8.6%) (Table 2). Notably, all of these changes were statistically significant when compared to pitavastatin alone. Pemafibrate as an add-on therapy resulted in a more anti-atherogenic lipoprotein profile, i.e., increment of cholesterol in medium, small, and very small HDL subclasses and in large and medium LDL subclasses [79].

In the second trial, pemafibrate (0.2 mg/day) was given for 24 weeks to 423 patients with residual dyslipidemia (TGs ranging from 325 to 333 mg/dL) on statins (most commonly atorvastatin, rosuvastatin and pitavastatin); LDL-C was around 108 mg/dL. Notably, if TGs were ≥ 150 mg/dL after 12 weeks, pemafibrate was up-titrated to 0.4 mg/dL. Regardless of statin background, combination therapy with pemafibrate 0.2 or 0.4 mg/day led to TG reductions of about 50% from baseline [79] (Table 2). Compared to the monotherapy arm, patients receiving pemafibrate showed a significant decrement in non-HDL, ApoB and ApoCIII, as well as an increment in HDL-C, ApoAI and ApoAII [79]. As previously described, the addition of pemafibrate led to an increment in medium and small lipid-poor HDL, more efficient in reverse cholesterol efflux [80]. In both of these last two studies, the incidence of adverse events during treatment was similar across all groups. The proportion of patients experiencing elevated alanine transaminase (ALT), creatine kinase (CK) and serum creatinine were comparable.

The clear definition of efficacy of pemafibrate on the lipid profile, i.e., TG reduction and HDL-C increment, in preclinical studies as well as in phase 1 and phase 2 clinical trials (previously reviewed [73,81]) led to the planning of the PROMINENT (pemafibrate to reduce cardiovascular outcomes by reducing triglycerides in patients with diabetes) trial (registered as NCT03071692). The primary objective of this phase 3 study is evaluation in T2D patients already on statin (fasting TGs: ≥200 to <500 mg/dL; HDL-C ≤ 40 mg/dL), testing whether pemafibrate (0.2 mg bid) can delay the time of the first occurrence of nonfatal myocardial infarction (MI), nonfatal ischemic stroke, hospitalization for unstable angina requiring unplanned coronary revascularization, and CV death. Changes in lipid end-points including ApoAI, ApoCIII, ApoE and non-fasting remnant cholesterol are listed as secondary outcomes [82].

The efficacy of fibrates on CV prevention has been disputed, mainly on the ground of studies on non-selected patients [63,64]. Long-term re-evaluation of some of the most recent trials has clearly confirmed that fenofibrate in particular may have a valid indication in reducing CVD in patients with diabetes, hypertriglyceridemia and low HDL-C [65]. The recently available low-dosage pemafibrate seems to provide a higher activity on the HDL system and the ongoing studies on vascular prevention will provide further data on the link between biochemical markers of the MetS and CV risk.

### 3.2. Omega-3

Fatty acids of the n-3 series (i.e., with multiple double bonds, the first one being in the n-3 position from the terminal methyl group) have provided an important addition to the dietary treatment in syndromes characterized by elevated TGs. Omega-3s act as “fraudulent fatty acids” [83] i.e., they, somewhat similar to drugs with a fatty acid-like structure, particularly fibrates, do not enter the liver metabolic handling by the classical fatty acetylCoA oxidative mechanism, with carnitine mediated transport to mitochondria [84]. They are instead handled by a non-mitochondrial regulated pathway, differently expressed both in the liver and other tissues [85]. Peroxisomal associated receptors are stimulated in the presence of those fatty acids catabolized not only by the classical mitochondrial pathway [86]. Both fibrates and omega-3s, thus, will not act as classical substrates of mitochondrial metabolism, but rather stimulate the metabolism of fatty acids coming from diet or end products of, e.g., TG metabolism by the PPAR-α mediated pathway. Peroxisomal stimulation is less extensive than in the case of fibrates, but it can well stimulate fatty acid oxidation. An additional mechanism, more closely related to the plasma glucose elevation in MetS, is the activation of tissue glucose uptake by the GLUT4 transporter in adipocytes; this mechanism appears to be mediated by the GPR120 protein, functioning as an omega-3 fatty acid receptor/sensor [87].

Controlled trials in patients given relatively elevated daily doses of omega-3 in the form of TGs or, more recently, of ethyl esters of eicosapentaenoic (EPA) and docosahexaeneoic (DHA) acids, as well as with novel formulations of separate fatty acids [88], have repeatedly confirmed a TG reduction in hypertriglyceridemic conditions associated or not with diabetes [89,90]. Controlled studies indicate lowering, in general, of 20–30% of fasting triglyceridemia in these conditions [91], with a moderate rise of HDL cholesterolemia as well as an increment in reverse cholesterol transport mainly by influencing HDL remodeling and promoting hepatobiliary sterol excretion [92].

A general review on the mechanism of omega-3, improving abnormalities characteristic of MetS, may in addition to the classical activation of fatty acid metabolism involve increased adipocyte differentiation, reduced lipolysis and lipogenesis, as well as a significant activity on low grade inflammation, including reduced adipokines and specialized pro-resolving lipid mediators [93]. By the activation of fat metabolism and consequent energy expenditure, it being peroxisomal and to some extent also mitochondrial, positive effects on obesity may be observed. Indeed, PPAR-α KO-obese mice show, in fact, a clear worsening of obesity that may be instead improved by omega-3 administration in different diet-induced conditions [94]. Reduced lipogenesis and low-grade inflammation may be of value in the treatment of complex metabolic disorders.

In the case of adipose tissue biology, the most recent evidence points out to the importance of both white and brown adipose tissue (WAT and BAT) function. “Healthy adipocytes” in WAT are relatively small fat cells with a high capacity for mitochondrial oxidative phosphorylation, TG/FA cycling and de novo lipogenesis [94]. These cells, with a flexible phenotype, may provide beneficial local and systemic effects by protecting against inflammatory responses during lipolysis, preventing fat accumulation and dyslipidemia caused by increased liver VLDL-TG synthesis.

Indeed, dietary omega-3s may indeed redirect adipose tissue to a “healthy phenotype” [94]. These polyunsaturated fatty acids appear to stimulate the “G protein coupled receptor” GPR120 [95], promoting BAT activation [96], thus inducing brown as well as beige adipocyte differentiation and thermogenic activation which seems to be linked to an increase in blood FGF-21 levels. Characteristically, in animals devoid of GPR120, adipose tissue thermogenic activation is not achieved [97]. These observations are of ethnological significance, in view of the high consumption of omega-3 from fish in individuals living in cold areas such as the Eskimos [98]. Interestingly, in in vitro systems only BAT and not WAT cells synthesize DHA [99].

Inflammatory changes in the adipose tissue are characteristic of obesity. They are driven by rises in circulating endotoxins and infiltrate immune cell populations. This will lead to an increased secretion of inflammatory adipokines (e.g., IL-6, TNF–α, monocyte chemoattractant protein (MCP) and chemokine (C-C motif) ligand (CCL) from multiple cellular sources [100]. The end result of the increased secretion of inflammatory mediators is the development of insulin resistance. A characteristic reduction in chemokine secretion from LPS stimulated co-cultures of omega-3 fed rodents is followed by a reduced secretion of IL-6 (−42%) and TNF-α (−67%), as well as by a similar reduction of other inflammatory mediators. Concomitantly, omega-3s increase the mRNA expression of negative regulators of inflammatory signaling, such as the monocyte chemoattractant 1-induced protein (MCPIP; +9.3-fold) and the suppressor of cytokine signaling 3 (SOCS3; +1.7-fold) [101]. In patients with MetS a reduction of high sensitivity CRP levels [102] has been found, although data from a number of clinical studies on this topic have been inconsistent [91,103].

It can thus be concluded that, in the adipose tissue, besides the stimulation of fatty acid catabolism, well-characterized anti-inflammatory and anti-chemotactic effects can be exerted by omega-3s. All these effects, recognized at the cellular level, may be followed by biochemical changes in patients with MetS and diabetes mellitus. In these patients, statistically significant TG reductions, compared to placebo, have been observed with, however, somewhat differential effects on LDL-C. Apparently, products containing DHA may increase LDL-C levels, whereas those containing EPA only products do not lead to a similar consequence [104,105].

#### Omega-3: Evidence from the Most Recent Clinical Trials

Most recently, the effect of omega-3 fatty acids (2 g daily) in reducing TGs and other lipid concentrations in patients with severe hypertriglyceridemia (TG > 500 mg/dL and <2500 mg/dL) was evaluated in the EVOLVEII (Epanova^®^ for lowering very high triglycerides II) trial, a double-blind, randomized, olive oil-controlled study. After an 8-week screening period for patients who required washout or stabilization of lipid-lowering therapy (e.g., statin or cholesterol-absorption inhibitors), they were randomized to receive two 1 g soft gelatin capsules with omega-3 (550 mg EPA + approximately 200 mg DHA in a new formulation) or olive oil once a day for 12 weeks. Notably, stratification was carried out based on TG levels, i.e., ≥ 500 ≤ 885 mg/dL or > 885 < 2500 mg/dL. Omega-3 capsules reduced TG by −28.1% vs. −10.2% (olive oil) in the group with TGs ≥ 500 and by −37.5% vs. −9.3% (olive oil) in the group with TGs > 885 mg/dL. In the whole population, TG differences between the two treatment groups were −14.2%. Non-HDL-C percentage changes were instead −8.8% (omega-3) vs. +0.4% (olive oil) in the group with TGs ≥ 500, with more marked differences in those with TG > 885 (−14% vs. +3.1%), and an overall −9% non-HDL-C reduction (Table 2). Omega-3 supplementation led to a significant lowering of VLDL-C, both when compared to baseline or to the olive oil arm. The decrease of VLDL-C concentrations was similar to that of TGs. HDL-C were modestly raised by both treatments, with no extra benefit given by omega-3 [106].

The results of this trial are in line with those found in the previous EVOLVE (Epanova for lowering very high triglycerides) double-blind, randomized, parallel, 4-arm study. In subjects with severe hypertriglyceridemia (TGs ≥ 500 mg/dL but <2000 mg/dL), administration of omega-3-FA 2 g/die (plus olive oil 2 g/day), omega-3FA 3 g/die (plus olive oil 1 g/day), or omega-3-FA 4 g/day for 12 weeks in combination with diet and lifestyle changes led to a −31% reduction in fasting TG in the group receiving omega-3-FFA 4 g/die vs. 25% in the other two treatment groups. A minimal TG reduction (−4.3%) was found in patients receiving olive oil 4g/day. A similar trend was found for non-HDL-C, with a maximal −9.6% reduction with omega-3-FA 4 g/day, vs. a +2.5% increment in the olive oil group (Table 2). HDL-C were not significantly changed at any dosage [107].

The effect of omega-3 as an add-on therapy to a statin background was evaluated in the ESPRIT (Epanova combined with a statin in patients with hypertriglyceridemia to reduce non-HDL cholesterol) trial, on persistently hypertriglyceridemic patients already on a maximally tolerated dose of statin or statin + ezetimibe, with TG levels ≥ 200 mg/dL and <500 mg/dL). Compared to olive oil (4 g/day), omega-3-FA 2 g/day or omega-3-FA 4 g/day administration led to a significant reduction in non-HDL-C (−3.9% and −6.9%, respectively) and TG (−14.6% and −20.6%, respectively) (Table 2) [108].

A limited number of studies have selectively evaluated the efficacy of omega-3 fatty acids on CV outcomes, both in primary and secondary prevention. Positive outcomes were reported from the GISSI (Gruppo Italiano per lo studio della sopravvivenza nell’infarto miocardico) [109] study which dealt mainly with patients with an acute coronary syndrome, and from the JELIS (Japan eicosapentaenoic acid lipid intervention study) [110] testing the efficacy of omega-3 (1800 mg/day) in primary prevention moderately hypercholesterolemic patients, mainly on statins. In contrast, the large alpha omega trial on coronary patients on a smaller daily intake of EPA + DHA (400 mg/day) failed to reach the targeted reduction of CV events [111]. While these last authors, in a recent meta-analysis on 77,917 patients in 10 different studies, appeared to confirm the lack of a significant impact of omega-3 on CV endpoints [112], at present, the effect of omega-3 supplementation on CV outcomes, i.e., any component of the composite of major adverse cardiac events (MACE), is being evaluated in the STRENGTH (study to assess statin residual risk reduction with Epanova in high cardiovascular risk patients with hypertriglyceridemia) and REDUCE-IT (reduction of cardiovascular events with icosapent ethyl–intervention) trials [113] targeting particularly hypertriglyceridemic patients.

Unquestionably, the efficacy of omega-3 intake on metabolic parameters and on inflammatory changes is now well established, with a convincing series of mechanistic studies, but the efficacy of these nutritional supplements on CV outcomes is at present unsettled. The ongoing studies should shed light on this last issue, particularly as pertains to patients with hypertriglyceridemia associated risk.

## 4. PPAR-γ Agonists

Agonists of PPAR-γ belong to the thiazolidinedione (TZDs) drug class and are currently in use for T2D [114]. Pioglitazone and rosiglitazone are the only two drugs available. Following epidemiological data indicative of a raised CV risk after rosiglitazone [115,116], the drug was taken off the market in Europe. Pioglitazone appears instead to reduce CV events [117]. Clinical trials with pioglitazone, e.g., PERISCOPE, PROactive and CHICAGO, have, in fact, demonstrated that in addition to beneficial effects in reducing TGs and increasing HDL-C levels, pioglitazone can reduce CV risk in T2D patients [118].

The PERISCOPE trial (pioglitazone effect on regression of intravascular sonographic coronary obstruction prospective evaluation) recruited patients with baseline HbA1c ≥ 6.0% to ≤9.0% (if on a glucose-lowering medication) or ≥6.5% to ≤10% (if not on drug therapy) with positive coronary angiogram (at least 1 angiographic stenosis with at least 20% narrowing). Pioglitazone, compared with glimepiride, raised HDL-C by +5.7 mg/dL vs. 0.9 mg/dL, whereas TG levels were decreased by −16.3 mg/dL vs. a rise of +3.3mg/dL. Fasting insulin levels were decreased by pioglitazone and raised by glimepiride. The primary end-point, namely the percent atheroma volume change measured by intravascular ultrasound (IVUS), was reduced by −0.16% in the pioglitazone arm vs. a +0.73% rise in the glimepiride group (Table 2); these between group changes were statistically significant [119]. Interestingly, a post-hoc analysis showed a greater relative increase in HDL-C (+14.2% vs. 7.8%) with a concomitant relative reduction of TGs (−13.3% vs. −1.9%), TG/HDL-C ratio (−22.5% vs. −9.9%) and HbA1C (−0.6% vs. −0.3%) upon pioglitazone administration, possibly responsible for the atheroma regression [120].

The CHICAGO (carotid intima-media thickness in atherosclerosis using pioglitazone) trial tested the hypothesis that pioglitazone would have a beneficial effect in reducing carotid intima-media thickness (IMT) progression compared with glimepiride [121]. The reported reduced progression of IMT appeared to be associated with a rise of HDL-C (+14%) following pioglitazone (Table 2) [122]. The positive effect of pioglitazone on carotid IMT is thus independent of its glucose lowering effect. Of note, weight and body mass index were higher in the pioglitazone arm.

The IRIS (insulin resistance intervention after stroke) trial showed that pioglitazone reduced the occurrence of fatal and non-fatal stroke or MI in insulin resistant patients without diabetes, also halving the occurrence of diabetes (Table 2). Notably, this latter occurred in 3.8% of patients on pioglitazone vs. 7.7% of those assigned to placebo (hazard ratio: 0.48; 95% CI: 0.33–0.69) [123,124]. Evaluation of safety outcomes indicated that pioglitazone led to (i) a weight gain of 4.5 kg (52.2% of patients) vs. +13.6 kg (11.4% of patients) for placebo [123]; with (ii) an increment in the absolute risk of fractures risk by 1.6% vs. 4.9%, depending on fracture classification [125]; and (iii) a higher incidence of edema (+35.6% vs. +24.9%) [123], this last being an as yet poorly understood frequent side effect of glitazones [126].

The clinical outcome studies on PPAR-γ agonists have been focused mainly on pioglitazone, particularly in view of the better tolerability. These have concluded that this treatment may be associated both with a reduced atheroma progression and a lower incidence of diabetes. Further, the IRIS study provided clear evidence of the efficacy of pioglitazone in preventing CV outcomes in insulin resistant patients [123,124].

## 5. PPAR Dual Agonists

Dual PPAR agonists or partial agonists, e.g., dual α/γ, α/δ or β/δ [127] were developed with the aim of achieving the TG-lowering and HDL-raising effects of PPAR-α activators as well as the insulin-sensitizing and antihyperglycemic effects of TZDs with a single drug. Such a combination of effects would be ideal for the treatment of T2D, MetS and NAFLD, which all share as common features atherogenic dyslipidemia and insulin resistance [128].

Of particular interest is the case of NAFLD, for which dual PPAR-α/δ agonists offer significant hope. Elafibranor (formerly known as GFT-505), with preferential α (EC50 = 6 nM) and complementary δ (EC50 = 47 nM) receptor agonist activity, is targeted to the liver, where it is converted to the main active metabolite, GFT-1007, in a dose-dependent manner. Elafibranor has been shown to be effective in disease models of NAFLD/NASH and liver fibrosis [129], as well as in T2D patients for whom a reduction in TG and LDL-C levels and improved insulin sensitivity were reported [130]. Recent results from the Phase 2b GOLDEN trial (NCT01694849) indicated that elafibranor treatment leads to a substantial histological improvement of NASH, including resolution of steatohepatitis and reduced CV risk. NASH was resolved without fibrosis worsening in 23% and 21% of patients assigned to receive either 80 mg or 120 mg/day elafibranor vs. 17% in the placebo arm; no significant differences between groups were found. When a more stringent definition of NASH was considered, changes in NASH resolution were 19% after elafibranor administration vs. 12% in the placebo group (*p* = 0.045) (Table 2) [131].

For activators of PPAR α + γ receptor, only preliminary data are available for saroglitazar, i.e., positive effects on the lipid profile, blood pressure, atherosclerosis, inflammation, and clotting [132]. Saroglitazar is being tested in an ongoing phase 3 trial in non-cirrhotic biopsy-proven NASH patients, in order to evaluate a possible improvement in NASH histology without worsening of fibrosis [133]. Interestingly, this agent is currently approved in India for the treatment of diabetic dyslipidemia [134]. Other glitazars, i.e., tesaglitazar and rasaglitazar have been discontinued from clinical development due to renal side effects, anaemia and leukopenia (tesaglitazar) and bladder tumor development (ragaglitazar) [135].

At present, dual agonists show an attractive metabolic profile, including an activity on MetS associated liver abnormalities. Clinical outcome studies are awaited with interest.

## 6. Conclusions

The management of MetS represents one of the major targets in atherosclerosis prevention. While treatment of hypercholesterolemia or diabetes can be successfully handled with drugs targeting cholesterol biosynthesis or beta-islet-cell function, MetS is characterized by a number of diverse metabolic abnormalities which are more difficult to pursue. While lifestyle modifications [136], including changes in diet and increased exercise, can provide help to a small number of patients, an improved knowledge of drugs affecting PPAR system has led to more frequent and better focused treatment choices. PPAR agonist “fraudulent fatty acids”, i.e., fibrates and omega-3 fatty acids, find a growing role in the handling of hypertriglyceridemia and, in the case of fibrates, also positively affecting HDL-cholesterol and the consequently raised CV risk. PPAR-γ agonists are instead targeted to the glycemic abnormalities of MetS; they may, however, lead to weight increase. The causal role of hypertriglyceridemia as a CV risk factor, confirmed by Mendelian randomization studies, has brought clinicians back to this somewhat forgotten risk marker, now rated by many as an unmet need [137]. The HDL-C raising approach has also become of high interest after the preventive failure of drugs such as the cholesteryl ester transfer protein inhibitors [138], disclosing the as yet not fully clarified CV protective mechanism of HDL [139].

## Figures and Tables

**Table 1:** Risk factors for the clinical diagnosis of metabolic syndrome.

	Value	Alternative Indicator
Waist circumference	* >94 cm in males, >80 cm in females ** >102 cm in males, >88 cm in females	
Raised blood pressure	Systolic ≥130 and/or diastolic ≥85 mm Hg	Treatment of previously diagnosed hypertension
Raised FPG	≥100 mg/dL (5.6 mmol/L)	Previously diagnosed of T2DM
Raised TG	>150 mg/dL (1.7 mmol/L)	Specific pharmacological treatment
Reduced HDL-C	<40 mg/dL (1.0 mmol/L) in males <50 mg/dL (1.3 mmol/L) in females	Specific pharmacological treatment

* Based on the International Diabetes Federation (IDF) threshold for Europid population. ** Based on the AHA/NHLBI (ATP III) threshold for USA population. FPG, Fasting Plasma Glucose; TG, triglycerides; HDL-C, High-Density Lipoprotein-Cholesterol; T2DM, Type 2 Diabetes Mellitus. Conversion factors: (i) mg/dL cholesterol = mmol/L × 38.6; (ii) mg/dL triglycerides = mmol/L × 88.5 and (iii) mg/dL glucose = mmol/L × 18. Reproduced with permission [8].

**Table 2:** Effect of PPARs on the features of metabolic syndrome—evidence from clinical trials.

PPAR-α Agonist	Clinic Study	Major Findings
*Pemafibrate*	Phase 3 (JapicCTI-142412; clinicaltrials.jp) follow-up: 24 weeks subjects: 166 [74]	1. Reduction in TGs: −45% 2. Decrement in non-HDL 3. Increase in HDL cholesterol
	Phase 3 (JapicCTI-142620; clinicaltrials.jp) follow-up: 24 weeks subjects: 225 [76]	1. Reduction in TGs: −46.2% 2. A further −6.5% TG reduction compared to fenofibrate
	Phase 3 (JapicCTI-121764; clinicaltrials.jp) follow-up: 12 weeks subjects: 489 [77]	1. TGs: −46.3% (0.1 mg/day), −46.7% (0.2 mg/day) and −51.8% (0.4 mg/day) vs. −38.3% (fenofibrate 100 mg/day) and −51.5% (fenofibrate 200 mg/day)
	Phase 2 follow-up: 12 weeks subjects: 188 [79]	1. Reduction in TGs: range from −46.1% to −53.4%
	Phase 2 follow-up: 24 weeks subjects: 423 [79]	1. Reduction in TGs: range from −46.8% to −50.8%
	On going Phase 3 trial PROMINENT (Pemafibrate to Reduce Cardiovascular OutcoMes by Reducing Triglycerides IN patiENts with diabeTes)—NCT03071692	Outcomes: First occurrence of nonfatal myocardial infarction, nonfatal ischemic stroke, hospitalization for unstable angina requiring unplanned coronary revascularization, and CV death.
*Omega-3*	EVOLVEII (Epanova^®^ for Lowering Very High Triglycerides II)—NCT02009865 Phase 3 follow-up: 12 weeks subjects: 162 [106]	1. Reduction in TGs: −14.2% 2. Reduction in non-HDL-C: −9%
	EVOLVE (The EpanoVa fOr Lowering Very high triglyceridEs)—NCT01242527 Phase 3 follow-up: 12 weeks subjects: 399 [107]	1. Reduction in TGs: range −25.5%/−30.9% 2. Reduction in non-HDL-C: range from −6.9% to −9.6%
	ESPRIT (EPANOVA Combined with a STATIN in PATIENTS With HYPERTRIGLYCERIDEMIA to Reduce Non-HDL CHOLESTEROL)—NCT01408303. Phase 3 follow-up: 6 weeks subjects: 647 [108]	1. Reduction in TGs: range from −14.6% to −20.6% 2. Reduction in non-HDL-C: range from −3.9% to −6.9%
	On going phase 3 trials: (i) STRENGTH (Study to assess statin residual risk Reduction with Epanova in high cardiovascular risk patients with Hypertriglyceridemia)—NCT02104817 (ii) REDUCE-IT (Reduction of Cardiovascular Events with Icosapent Ethyl–Intervention)—NCT01492361 [113]	Outcomes: First occurrence of cardiovascular death, nonfatal MI, nonfatal stroke, emergent/elective coronary revascularization, or hospitalization for unstable angina
*Pioglitazone*	The PERISCOPE Trial (Pioglitazone Effect on Regression of Intravascular Sonographic Coronary Obstruction Prospective Evaluation)—NCT00225277 Phase 3 follow-up: 18 months subjects: 543 [119]	1. Percent atheroma volume change: −0.16% 2. Raise in HDL-C: +5.7 mg/dL 3. Decrement in TGs: −16.3 mg/dL
	The CHICAGO (Carotid Intima-Media Thickness in Atherosclerosis Using Pioglitazone) trial—NCT00225264 Phase 3 follow-up: 72 weeks subjects: 462 [121,122]	1. Progression of mean CIMT: −0.013 mm vs. glimepiride 2. Progression of maximum CIMT: −0.024 mm vs. glimepiride 3. HDL-C: +14%
	The IRIS (Insulin Resistance Intervention after Stroke)—NCT00091949 Phase 3 follow-up: 4.8 years subjects: 3876 [123,124]	1. Reduction of stroke or MI in insulin resistant patients 2. Reduction in recurrence of diabetes: −52%
*Elafibranor*	GOLDEN trial—NCT01694849 Phase 2b follow-up: 52 weeks subjects: 256 [131]	1. NASH resolution in 19% of patients

All percentage changes are vs. baseline otherwise differently indicated. CIMT, Carotid Intima-Media Thickness; CV, cardiovascular; HDL-C, high-density lipoprotein cholesterol; MI, Miocardial Infarction; NASH, nonalcoholic steatohepatitis; TG, triglyceride.
